# NEWS

**DOI:** 10.7189/jogh.04.020201

**Published:** 2014-12

## AFRICA

➢ Aliko Dangote, Africa’s wealthiest man, has pledged to build 11 health centres in Kano in north–west Nigeria, in an effort to improve routine immunisation and health for the state’s citizens. Kano, with significant numbers of under–immunised children, has been particularly vulnerable to polio, and Nigeria remains one of three countries where polio is endemic. The Kano state government has already signed an agreement with the Bill and Melinda Gates Foundation to support free routine immunisations. Mr Dangote said he was minded to build the new centres because of the state government’s commitment to better healthcare services, and he assured the governor that his foundation will work with them to strengthen the state’s immunisation programme. (*Forbes*, 3 Jul 2014)

➢ Zambian sex workers report that efforts to reduce HIV infections are hampered by demand for unprotected sex, often at a price premium. This is increasing as circumcised men believe they cannot contract HIV and sexually transmitted infections. The Zambian Centre for Infectious Disease states that male circumcision provides 60% protection against HIV in line with WHO recommendations, along with other preventative measures. Sex workers are increasingly knowledgeable on health risks, and are supplying additional condoms if their client does not have any. Some of Zambia’s sex workers are joining the Lifestyle Health Foundation, which campaigns to raise awareness on the dangers of unprotected sex. (*allafrica.com*, 11 Jul 2014)

➢ Ghana’s 10–year–old National Health Insurance Scheme (NHIS) provides healthcare access, has been awarded the UN Global Award for Excellence, and countries such as Ethiopia, Benin, Mali and Bangladesh have visited Ghana to learn from it. 70% of its registrants pay no premiums, as certain categories (e.g. pregnant women, the elderly, disabled people, etc.) are exempt. However, 15 million from Ghana’s 25 million population are unregistered and citizens report problems with registration and accessing healthcare. Registration queues can begin at 3 am with no guarantee of success – a huge burden on families and ill people. Others report having to wait 6–8 hours to see a doctor, and concerns over substandard medicines. The NHIS is taking steps to improve access, although Ghana remains seriously short of health professionals, and many Ghanaians continue the “cash and carry” system of healthcare access. (*Al Jazeera*, 6 Aug 2014)

➢ Nigeria met with its first Ebola case in July, followed by an additional scattering of cases across two states. However, there have been no new cases since 5 September 2014, and this success offers valuable lessons in dealing with an Ebola outbreak. According to a report published in *Eurosurveillance*, the key elements were: fast and thorough tracing of all potential contacts; ongoing monitoring of them; and rapid isolation of potentially infectious contacts. With a centralised and co–ordinated Incident Management System, Nigeria’s authorities identified cases quickly, and rigorously surveyed their contacts, thus preventing Ebola’s further spread. Nigeria could draw on funding, staff and tools to halt Ebola’s spread; but in the three countries worst affected by Ebola, implementing standard outbreak control procedures are near–impossible due to dire infrastructure and capacity. (*Scientific American*, 18 Oct 2014)

➢ The World Bank will invest US$ 100 million in increasing the number of overseas health workers in West Africa to care for people with Ebola. Doctors, nurses and other healthcare workers are desperately needed to staff treatment centres – according to the UN, 5000 international workers are needed, including 1000 health workers. There has been a gap in providing training to healthcare workers, due to safety concerns over the risks of contracting Ebola, and workers have sought reassurance that they would be repatriated in the event of illness. The new funds will set up coordination hubs with the affected countries’ governments, the WHO, the UN’s Ebola co–ordination centres and other agencies to recruit, train and deploy qualified health workers. (*The Guardian*, 30 Oct 2014)

## ASIA

➢ Drug–resistant malaria parasites have spread to border regions of Southeast Asia, threatening efforts to control and eliminate the disease. WHO states that resistance to the most effective drug, artemisinin, is reported in four countries, and 64 countries have evidence of resistance. There are no signs of resistance in Kenya, Nigeria and DR Congo, but if resistance spreads out of Asia and into Africa much progress in decreasing malarial deaths will be reversed. Although new drugs and a vaccine are in the pipeline, they are several years from market so existing drugs must be conserved where they are still working. Prof. Nicholas White, chair of the Worldwide Antimalarial Resistance Network, says that conventional malaria control measures will not be enough, calling for it to be a global public health priority. (*Reuters*, 30 Jul 2014)

➢ The Indonesian government has launched a programme to promote safer cooking practices, which aims to prevent 165.000 premature deaths each year. It will introduce affordable, biomass–fuelled, cooking stoves to the 24.5 million households who still use traditional (mostly firewood) methods for cooking. Household air pollution from firewood stoves increases the risks of asthma, lung TB and acute respiratory infections, especially in children who spend more time indoors. The programme will include a public awareness campaign of the risks of firewood. The World Bank has signed agreements with the Indonesian government and PT Bank Rakyat Indonesia to support the programme’s second phase. (*World Bank*, 14 Aug 2014)

➢ Vietnam’s Ministry of Finance is proposing a phased increase consumption tax on cigarettes from its current 42% to 65% in 2014, 75% in 2015 and 85% in 2015. This would raise US$ 136.5 million of revenue in 2015, rising to Ł362.5 million in 2018. The WHO estimates that if additional taxes increase the price of cigarettes by 10%, consumption would fall by 5%. According to the Ministry of Health, 15 million Vietnamese people smoke – one of the highest rates in the world – and there are 40 000 tobacco–related deaths each year, set to rise to 70 000 per year by 2030. There are concerns that the proposed tax increases are insufficient to decrease consumption, given that cigarettes are relatively cheap in Vietnam. (*Xinhua*, 16 Sep 2014)

➢ Historically, Indonesia has a uniquely strong and successful family planning programmes, doubling its contraceptive prevalence rate to almost 60% from 1976–2002, and halving its fertility rate to 2.6 children per woman. This helped towards Indonesia’s impressive annual economic growth rate of 5%. However, progress has stalled, mainly due to shifting responsibilities for these programmes, with resulting uncertainties over roles. The Indonesian government is attempting to revitalise its family planning programmes, by strengthening local programmes to bolster access, free service access, and improved health worker training and facilities etc. Ultimately, the government aims to reduce fertility to 2.1 children per woman by 2015. (*Devex*, 25 Sep 2014)

➢ In Afghanistan, opioid use and dependency were the main contributors to disability, morbidity and mortality (e.g. via overdoses, HIV infection) from illicit drug use in 2010. Afghanistan has a tradition of opium smoking, and is a source of illegal opiates. More recently, Afghans have begun to inject heroin and use pharmaceutical opioids. It is estimated that 5.1% of the population have recently used drugs, and that this is increasing. This is a major problem in a war–scarred country with little treatment infrastructure. There is an urgent need for effective interventions such as substitution therapy, needle and syringe programmes to reduce the transmission of blood–borne viruses, and HIV and hepatitis C treatment programmes to reduce the infection burden. This needs additional resources and the co–ordinated implementation of programmes by the government and civil society, supported by international and non–governmental organisations. (*The Lancet Global Health*, 31 Oct 2014)

## AUSTRALIA AND WESTERN PACIFIC

➢ A survey found that 24% of girls and 34% of boys in the remote Cook Islands – dependent on imports for 82% of foodstuffs – are obese. The traditional diet of fish, fruit and taro has been replaced with imported, calorie–rich and nutrient–poor processed food and drinks, and the tourism boom has increased fast–food outlets. The Islands’ Director of Public Health, Dr Rangi Fariu, says that 80–90% of men are obese, with high cholesterol levels, blood pressure, diabetes and heart disease, and are dying prematurely as a result. To tackle this, the government has increased sugary drink taxes, and instigated an “invest in your health” campaign to encourage exercise, and fruit and vegetable consumption. However, it is a challenge to supply local produce to compete with processed imports, and the Ministry of Agriculture is working with the UN Food and Agriculture Organization to increase availability. (*The Guardian*, 2 Sep 2014)

➢ The New Zealand Ministry of Health has clamped down on the sale of e–cigarettes containing nicotine – previously readily available in shops – and have dispatched enforcement officers to inform retailers that their sale is prohibited. Leading public health specialists have criticised the move, noting that it could drive people back to traditional, more harmful, cigarettes, and half of the country’s smokers will die from smoking–related causes. The Ministry of Health recommends other cessation aids (e.g. patches and gum) rather than e–cigarettes. The WHO reports that e–cigarettes are less toxic that combustible cigarettes, but they do pose threats to adolescents and unborn babies, and increase exposure to second–hand nicotine and other toxicants. (*New Zealand Herald*, 14 Sep 2014)

➢ Comparisons between energy expenditure of today’s children with children in 1919 suggest that the latter were 50% more active than today’s children, with four more hours of physical activity and three less hours of sitting. In Australia, 25% of children now walk or cycle to school, compared to 70% in 1970, and the number of sports played by children is declined, as is the amount of outdoor free play (e.g. tree climbing). Australia’s children are in the bottom one–third of childhood fitness, and its children spend the third highest amount of time with TV and computer screens. This reflects a wider societal move towards less energy expenditure through labour–saving devices and sedentary work, with related weight increases. The challenge is offset these developments by creating spaces and technologies that encourage active leisure. (*theconversation.com*, 21 Sep 2014)

➢ The 2014 Aid Transparency Index ranked Australia as 25th out of 68 aid providers (“fair”), down one place from 2013. The UN Development Programme was first, followed by the UK’s Department for International Development, the US’s Millennium Challenge Corporation, GAVI and the Asian Development Bank. Donor countries are assessed on their commitment to aid transparency, including transparency of organisation– and activity–level information. The Index commented on Australia’s inaccessible project–level information, and criticises it for its ‘unambitious’ aid transparency implementation, urging full implementation by the end of 2015. (*DevPolicy Blog*, 9 Oct 2014)

➢ Fiji will stop sending peace–keeping police officers to Liberia due to the Ebola crisis, where it has 27 officers serving with the UN Mission in Liberia. The serving officers are reported to be in good health, and all necessary precautions are in place to protect them. Fiji’s Ministry of Health has activated its communicable taskforce, and is meeting with other agencies to ensure it is fully prepared for the Ebola virus. (*Xinhau*, 15 Oct 2014)

## CHINA

➢ In 2013, China’s mortality rate for children under 5 years of age fell to 20% of its 1991 level, and maternal mortality fell by 71%; whilst children with Hepatitis B fell from 10% to less than 1%. WHO and the World Bank reported China’s exceptional progress in reducing child and infant mortality, underpinned by better care at birth and countrywide immunisation. Women giving birth in hospital are subsidised, thereby reducing complications (especially neonatal tetanus), and bringing inaccessible groups into the health system. 95% of children are vaccinated against measles, rubella and polio, and eight vaccinations were added to its portfolio (although measles outbreaks show that children can miss doses). China has a blanket approach to vaccination, effective when infectious diseases are rife. A testimony to China’s initial success is that a more sophisticated and targeted approach is now required (e.g. screening infants for suitability), as many diseases have been eradicated. (*The Economist*, 26 Jul 2014)

➢ Air pollution, smoking, obesity and accidents – especially road accidents – kill at least 4.7 million Chinese people each year, according to research published in *The Lancet*. It showed that China’s health has improved in many ways, e.g. life expectancy has increased from 40 years in 1950 to 76 years in 2011, and many infectious diseases have rapidly declined. However, the risk of premature death and illness from pollution, smoking, accidents and other lifestyle–related illness is at record levels. The authors say that China can learn from developed countries’ experiences of non–communicable diseases, and many risks can be lowered by effective interventions. (*AFP*, 29 Aug 2014)

➢ Air pollution regulations over the past decade in the city of Taiyuan have substantially improved its citizens’ health, leading to a more than 50% reduction in costs arising from disability and loss of life. Taiyuan is a major centre for energy production and metallurgy, and the provincial government implemented new environmental legislation to combat air pollution, including allowing the closure of polluters, setting emission standards and promoting energy efficiency. This led to concentrations of particulate matter falling by 50% from 2000 to 2010, resulting in 30 000 fewer disability–adjusted life years arising from 2810 fewer premature deaths, 31 810 fewer hospital admissions and 141 457 fewer outpatient admissions. This is consistent with research that demonstrates a link between air quality and childhood developmental scores. It makes a strong case for tighter regulation, as only three out of 74 cities monitored by the government meet minimum air standards. (*Asian Scientist*, 10 Sep 2014)

➢ According to research published in *The Lancet Diabetes and Endocrinology,* China has the largest number of people with diabetes, reaching epidemic proportions in the adult population. In 1980, less than 1% of adults had diabetes, but this rose to 12% in 2010, and estimates show that around 50% of adults are pre–diabetic, putting them at risk of diabetes and related illnesses. Of particular concern is that 70% of diabetic adults are undiagnosed, 25% have received treatment and it is controlled in only 40% of these cases, suggesting increases in related cardiovascular and kidney disease, cancer etc. The epidemic is caused by rapid economic development and urbanisation. It also seems that Chinese people are relatively susceptible to type 2 diabetes, which tends to develop at lower body mass index. (*sciencedaily.com*, 10 Sep 2014)

➢ China will dispatch a unit of the People’s Liberation Army to Liberia, in response to UN calls for intensified efforts to fight Ebola in West Africa. The US has led the drive to halt the spread of the disease, and China has faced criticism for not doing enough to help its trade partner. The unit will build a 100–bed treatment centre in Liberia, the first one in the three countries most affected by Ebola. This is the first time that China has deployed an entire unit of epidemic prevention forces and military medical staff to another country. In other measures to combat Ebola, China has donated US$ 123 million, dispatched health workers, and sent doses of an experimental drug. Lin Songtian, a government minister, said that China’s assistance would not stop until the Ebola epidemic is halted. (*Reuters*, 31 Oct 2014)

## EUROPE

➢ Health expenditure continued to fall in Greece, Italy, Portugal and Spain, the Czech Republic and Hungary, according to the 2014 edition of OECD *Health Statistics*. Greece’s health spending fell in real terms by 25% between 2009 and 2012, mainly due to public spending cuts. This is in contrast to the USA (2.1% increase), Korea (6% increase) and Mexico (8.5%). Spain, France, Denmark and the UK saw particularly sharp increases in the market share for cheaper generic drugs, with increases of 100%, 60%, 44% and 28% respectively. (*pharmatimes.com*, 1 Jul 2014)

➢ Britain has a long history of clinical trials, but these are increasingly moving overseas; the number of trials fell by 14% between 2005 and 2013 whilst its global market share fell sharply. Each day’s delay in getting a drug to market can cost a company up to US$ 10 million, and trials are moving to east Europe and China which have fewer hurdles. The main challenges are the very slow process for testing new treatments in Britain, and difficulties in recruiting subjects. Britain risks losing its share of a global industry worth US$ 51 billion a year and, more subtly, the erosion of clinical standards. However, there is potential for more large trials, where researchers can take advantage of Britain’s unique centralised patient data system to trawl NHS data to recruit subjects. If medical confidentiality can be resolved, Britain could continue to lead the world in clinical testing. (*The Economist*, 26 Jul 2014)

➢ The appointment of the new EU leadership team to sit alongside the EC President Jean–Claude Juncker has slowed down the work of European institutions, and a paralysed commission cannot respond to external crises, e.g. Ukraine, Gaza and Central African Republic. Devex have identified five global long–term challenges essential to European prosperity which require international and European action. Firstly growth must deliver jobs and sustainable livelihoods, both to Europe’s young people and globally people living in extreme poverty. Secondly, Europe must work towards an ambitious global climate agreement in 2015, and the transition towards a green economy. Thirdly, conflict and state fragility must be tackled in the longer–term. Fourthly, human rights, from political prisoners to gender inequality, must be supported on a wider scale. Finally, the EU must tackle poverty and inequality both at home and in the developing world. (*Devex*, 1 Sep 2014)

➢ Sweden will announce US$ 14 million of funding for a new agency – the International Land and Forest Tenure Facility – that will provide grants and expertise to help indigenous people and forest communities to secure land rights. It will be independent, and governed by representatives from indigenous peoples, community and civil–society groups, donors and business. Where indigenous and other groups claim customary ownership of land and forests that is not legally recognised, conflict can arise with governments and business. This can be costly for investors, local people and ecosystems, and recognizing land rights can be an effective way to implement forest–based carbon–mitigation schemes. (*Thomson Reuters Foundation*, 18 Sep 2014)

➢ HIV continues to spread in Europe despite improved treatment and prevention options, with 136 000 new cases diagnosed across Europe and Central Asia in 2013 – an 80% increase from 2004. Roughly 105 000 cases were reported in Eastern Europe and Central Asia (EECA), 29 000 in the European Union and European Economic Area (EU/EEA), and 2000 in other non–EU countries. New cases in EECA doubled from 2004, remaining constant in EU/EEA. This means that Europe has not reached the Millennium Development Goal of halting and reversing the spread of HIV/AIDS. The WHO Regional Office for Europe states that high–risk populations are not being effectively reached – the main cause of the continued spread – and prevention and control among men who have sex with men is the cornerstone of European HIV programmes. The WHO Regional Office and European Center for Disease Prevention and Control call for a strengthened and tailored response to curb the epidemic. (*WHO*, 27 Nov 2014)

## INDIA

➢ India’s pharmaceutical regulator has cut and capped prices of 108 drugs used for a variety of diseases, which will affect the profit margins of drug companies such as Sanofi SA. This coincides with efforts by India’s health ministry to widen the list of essential medicines that will be subject to a price cap. Price caps help provide affordable medicines for the 70% of India’s people living on less than US$ 2 a day, and the more–than–80% without health insurance. The companies affected did not respond immediately, but analysts believe it could lead to more controls in the longer–term. (*Reuters*, 14 Jul 2014)

➢ In a study published in *Geophysical Research Letters*, pollution from ground–level ozone, formed by emissions from vehicles, cooking stoves etc., damaged 6 million tonnes of India’s wheat, rice, soybean and cotton crops in 2005. Wheat and rice are major food sources in India, and cotton is a major commercial crop. This caused cumulative losses of US$ 1.29 billion, and destroyed crops that could have fed millions of people living below the poverty line. India currently has no air quality standards to protect agriculture from ground–level ozone pollution, and this research could help policymakers formulate them. (*Times of India*, 5 Sep 2014)

➢ Following on from the September flooding, Kashmir is in the midst of a health crisis, hospitals damaged by flood water, and people living in areas choked by putrid, infectious and sometimes impassable water. This is worsened by many residents choosing to stay in their homes, thus increasing the risk of cholera, hepatitis A and typhoid outbreaks, when Kashmir has little scope to treat them. The state health minister, Taj Mohiuddin, outlined plans to deal with the crisis, including mobile units to dispense medicine and chlorine tablets, and getting the state’s main hospitals operational quickly. Although there have been few cholera cases to date, there are fears that they will increase, and that there will be a rush of people visiting doctors once the roads are passable – with the government could be hard–pressed to respond. (*New York Times*, 19 Sep 2014)

➢ India’s recent efforts to reach out to Africa, supported by Africa’s emerging middle–class being a ready market for India’s ‘frugal innovation’ products, and Africa’s abundant natural resources, were set back by India’s cancellation of the largest–ever India–Africa summit. This was due to the risk of Ebola being brought from Africa. However, the risk was small as three countries are suffering from Ebola, not the entire continent. Despite this, India is generously assisting to relieve the outbreak. It is difficult to strike a balance between protecting against Ebola and avoiding damage to trade and relationships with Africa, and isolation will not avoid a pandemic. Continued support in the form of donations, equipment and expertise is more helpful, and may be the best way of preventing its spread elsewhere. (*The Economist*, 13 Oct 2014)

➢ India has offered “full co–operation” to Pakistan in eradicating polio, noting concern over its neighbour’s accounting for 85% of global polio cases. Health Minister Harsh Vardhan said that previous co–operative efforts made little progress. He praised Pakistan’s latest initiative against polio, stating that a similar model in India was highly successful. India risks a polio outbreak imported from Pakistan, and the government will replace oral vaccines with injectable ones in 2015. (*The Economic Times India*, 24 Oct 2014)

## THE AMERICAS

➢ Mapp Biopharmaceutical, a small US biotech company, was at the centre of a race to find a cure for Ebola, and an ethical debate over using unproven treatments. Mapp produces the experimental drug ZMapp, given to two US aid workers with Ebola, who appear to have responded. Whilst this raises hopes for a cure, there are questions over why it did not emerge until two Americans were affected, rather than 900 West Africans. Mapp replied that the drug is at an early stage, untested on humans, the aid workers’ employer had requested it, and they lack capacity to scale–up production. Also, there are few commercial incentives to develop drugs for a sporadic disease in one of Africa’s poorest regions. Without proper trials, ZMapp’s effectiveness is unclear, as the aid workers may have survived regardless; and WHO is convening medical ethicists to explore this. (*Financial Times*, 7 Aug 2014)

➢ A UN Development Programme report shows that poverty levels in Latin America and the Caribbean fell from 41.7% to 25.3% from 2000–2012, lifting more than 56 million people out of poverty. It warns that 200 million people – 37.8% of the population – are vulnerable, and calls for more investment in social protection programmes as the pace of social and economic progress slows. It calculates poverty as living on less than US$ 4/day, and also reveals that the proportion of people living on US$ 4–10/day rose by 3.4% over the same period. It singled out Bolivia and Peru for the greatest poverty reductions – 32.2% and 26.3% respectively – and praised progress in Chile and Argentina. However, poverty levels remained constant in Uruguay, Honduras and the Dominican Republic, and rose by 6.8% in Guatemala. (*BBC News*, 26 Aug 2014)

➢ Tegucigalpa, the capital of Honduras, is one of the world’s most violent cities, with high levels of murder, rape and abductions. Rape survivors often do not report rapes out of fears of retaliation and stigmatisation, and only a small fraction receive the support and care they need. In response, Médecins Sans Frontières (MSF) and the Honduran Ministry of Health set up a priority service in 2011 to provide emergency support to the victims of violence, including sexual violence. The emergency medical treatment includes post–exposure prophylaxis against HIV. However, this vital service is unable to prescribe the emergency contraceptive pill to prevent pregnancy following rape, as it has been banned in Honduras since 2009. MSF campaigns for its legalisation, so that rape victims need not fear an unwanted pregnancy or an unsafe abortion. (*MSF*, 28 Aug 2014)

➢ Harvard University received US$ 350 million from the Morningside Foundation for the School of Public Health – the largest donation in its history. The donation is unrestricted, and will be used for student financial support, new teaching facilities, and seed funding for ground–breaking research etc. Julio Frenk, the School’s dean said the donation would particularly be used to support research and training in: pandemics from malaria and Ebola to obesity and cancer; environmental health risks; poverty and humanitarian crises; and failing health systems. (*Reuters*, 8 Sep 2014)

➢ Type 2 Diabetes Mellitus (T2DM) comprises 90–95% of all US diabetes cases, and prevalence rose from 25.8 million in 2010 to 29.1 million in 2012, although mortality decreased over the same period. A CDC study published in *The Lancet Diabetes and Endocrinology* examined the lifetime risk, and life years’ lost, of T2DM. It found that the average 20–year old American male’s T2DM risk rose from 20% in 1985–1989 to 40% in 2000–2011, and female risk from 27% to 39%. Hispanic men and women, and black women, have the highest risk at 50%. Life years’ lost fell by 1.9 for both sexes, although the overall number of years lost rose by 50%, and years spent living with T2DM increased by 156% and 70% for men and women respectively, due to increased incidence. This will increase pressure on resources, and more effective lifestyle interventions are needed to reduce new cases. (*Medical News Today*, 13 Aug 2014)

